# Evidence of inflammatory immune signaling in chronic fatigue syndrome: A pilot study of gene expression in peripheral blood

**DOI:** 10.1186/1744-9081-4-44

**Published:** 2008-09-26

**Authors:** Anne L Aspler, Carly Bolshin, Suzanne D Vernon, Gordon Broderick

**Affiliations:** 1 Department of Medicine, Faculty of Medicine and Dentistry, University of Alberta, Edmonton, Alberta, T6G 2H7, Canada; 2 The CFIDS Association of America, Charlotte, North Carolina, 28222, USA

## Abstract

**Background:**

Genomic profiling of peripheral blood reveals altered immunity in chronic fatigue syndrome (CFS) however interpretation remains challenging without immune demographic context. The object of this work is to identify modulation of specific immune functional components and restructuring of co-expression networks characteristic of CFS using the quantitative genomics of peripheral blood.

**Methods:**

Gene sets were constructed a priori for CD4+ T cells, CD8+ T cells, CD19+ B cells, CD14+ monocytes and CD16+ neutrophils from published data. A group of 111 women were classified using empiric case definition (U.S. Centers for Disease Control and Prevention) and unsupervised latent cluster analysis (LCA). Microarray profiles of peripheral blood were analyzed for expression of leukocyte-specific gene sets and characteristic changes in co-expression identified from topological evaluation of linear correlation networks.

**Results:**

Median expression for a set of 6 genes preferentially up-regulated in CD19+ B cells was significantly lower in CFS (p = 0.01) due mainly to PTPRK and TSPAN3 expression. Although no other gene set was differentially expressed at p < 0.05, patterns of co-expression in each group differed markedly. Significant co-expression of CD14+ monocyte with CD16+ neutrophil (p = 0.01) and CD19+ B cell sets (p = 0.00) characterized CFS and fatigue phenotype groups. Also in CFS was a significant negative correlation between CD8+ and both CD19+ up-regulated (p = 0.02) and NK gene sets (p = 0.08). These patterns were absent in controls.

**Conclusion:**

Dissection of blood microarray profiles points to B cell dysfunction with coordinated immune activation supporting persistent inflammation and antibody-mediated NK cell modulation of T cell activity. This has clinical implications as the CD19+ genes identified could provide robust and biologically meaningful basis for the early detection and unambiguous phenotyping of CFS.

## Background

Chronic fatigue syndrome (CFS) is estimated to cost the American economy over $9 billion each year in lost productivity [1]. Among other components chronic immune cell dysfunction and activation has been demonstrated in CFS by several groups [2-4]. Though similar in terms of broad lymphocyte classes CFS and non-fatigued subjects can be readily distinguished when specific immune cell subsets are examined. For example Klimas et al. [2] report a significant expansion CD26+ (DPP-IV) activated T cells in CFS subjects. This multifunctional molecule plays a major role in the regulation, development, maturation and migration of T helper (Th) and natural killer (NK) cells as well as in B cell immunoglobulin switching [5]. Moreover abnormal expression of CD26+ is found in autoimmune diseases [6]. More recently CFS patients were also reported to have significantly fewer CD3+/CD25- T cells and significantly more CD20+/CD5+ B cells [7], a subset associated with auto-antibodies. Significantly fewer CD56+ NK cells were also observed in recent work by Racciatti et al. [8]. Though important, flow cytometry results such as these leave many questions regarding cellular state unanswered. Microarray profiling of gene expression on the other hand offers a glimpse of pathway activation in disease pathogenesis at molecular resolution. Microarray analysis of cDNA profiles in peripheral blood mononuclear cells (PBMC) have revealed altered expression in CFS of several immune genes [9,10] involved in response to oxidative stress, NK cell activity and elements of antigen processing. Instability in immune response and restructuring of immune cell signaling under exercise challenge has also been observed [11]. Unfortunately microarray profiling is commonly performed on mixed cell populations producing an average profile from which it is very difficult to dissect the contributions of relative cell abundance, cell activation state and cell-cell signaling. More importantly, this averaging can obscure significant changes in the state of minority cell sub-populations.

These challenges notwithstanding, a review of this evidence strongly suggests that CFS pathogenesis is likely to include a characteristic immunologic component in at least one subset of the patient population [12]. However the exact nature of this immunologic component remains the object of considerable debate at least in part because of an inability to cast gene expression profiles in the useful context of immune cell demographics. In an attempt to address this issue methods have been proposed to dissect global gene expression profiles into discrete elements assignable to biologic processes [13-15]. The assignment of genes to discrete modules or sets has been successful in several respects. A first contribution involves simply reducing the dimensionality of >55,000 gene expression measures to that of say 10 or so gene sets. The interpretability of results is further enhanced by associating sets with basic cellular functions. Finally the numerical robustness is greatly improved through the averaging of changes in expression over many genes. In addition gene sets are transportable across microarray platforms making it possible to compare studies based on different technologies.

In this work we explore the use of discrete gene sets in extracting useful information regarding immune dysfunction in CFS from gene expression profiles of mixed lymphocyte populations. In particular we construct gene sets that capture elements of abundance and activity assignable to specific immune cell subsets thereby facilitating direct integration with flow cytometry results. Data from a large population-based study of CFS [16] is then examined for changes of immune set expression across two separate CFS classification approaches. In addition, patterns of coordinated expression linking these immune sets were investigated using simple correlation networks. These networks were examined for shifts in topology and point to patterns of immune signaling in CFS that are consistent with chronic inflammation. These observations could constitute a signature of CFS or a component thereof.

## Methods

### Subjects and diagnostic classes

Recently a dataset for a 2-day in-hospital study of CFS in the general population of Wichita Kansas was made available [16]. Referred to as the Wichita Clinical study, this investigation included a highly comprehensive spectrum of detailed clinical and laboratory measures and PBMC expression profiles for 20,000 genes. From this dataset a final analysis group of 111 female subjects was obtained by excluding the few male subjects and subjects with confounding medical or psychiatric conditions. Subjects in this dataset were classified as CFS using the CDC Symptom Inventory, Multidimensional Fatigue Inventory (MFI) and Short Form 36 (SF-36) instruments [17,18]. This classification will be referred to as "empiric" and resulted in 39 CFS, 37 non- fatigued (NF), and 35 subjects with insufficient symptoms or fatigue (ISF). A second classification proposed by Vollmer-Conna and colleagues [19] used latent class analysis (LCA) of 440 clinical and biological measurements to delimit 5 fatigue classes, a non-fatigued class and 2 unassigned individuals. Obese subjects with prominent post-exercise fatigue, hypnoea and disturbed sleep formed Class 1. Reasonably healthy subjects with few symptoms, low depression scores and good sleep composed Class 2. Subjects in Class 3 resembled those in class 1 but also displayed low heart rate variability during sleep and low 24-hour cortisol levels. Class 4 was populated with healthier, less depressed individuals having restful sleep but suffering muscle pain. Finally Classes 5 and 6 both captured less obese but highly symptomatic and depressed individuals with prominent post-exercise fatigue. Individuals in Class 6 also displayed disturbed sleep with low heart rate variability and low cortisol. The patient demographics for each of these classification systems are summarized in Table 1 and the alignment between these systems is described in Table 2. The collection and processing of PBMCs including hybridization to MWG microarrays (MWG Biotech, Ebersberg, Germany) are described in Vernon and Reeves [16]. Details of the microarray data preprocessing including normalization, outlier detection and false discovery correction are available in Broderick et al. [9].

**Table 1:** Demographic data for 111 subjects from the Wichita clinical study

	**Empiric Classification**			**LCA Classification**					
		
	**CFS** (n = 39)	**IFS** (n = 35)	**Controls** (n = 37)	**LCA-1** (n = 23)	**LCA-3** (n = 17)	**LCA-4** (n = 11)	**LCA-5** (n = 14)	**LCA-6** (n = 11)	**LCA-0/2** (n = 35)
**Mean Age **(SD)	51.4 (8.2)	50.3 (8.2)	51.6 (9.0)	50.9 (7.6)	54.7 (5)	44.4 (8.7)	48.2 (10.2)	55.8 (3.4)	51.3 (9)
**Mean Years Ill **(SD)	16.7 (11.0)	14.4 (10.0)	2.8 (5.0)	15.5 (10.7)	11.3 (4)	16.0 (12.3)	16.8 (10.5)	16.4 11.2	14.3 (12.9)
**Race **[n (%) ]									
White	35 (90.0)	32 (91.4)	36 (97.3)	21 (91)	17 (100)	10 (90.9)	11 (78.6)	11 (100)	33 (94.3)
Black	1 (2.6)	3 (8.6)	1 (2.7)	1 (4.4)	0 (0)	1 (9.1)	1 (7.1)	0 (0)	2 (5.7)
Multiple Race	2 (5.1)	0 (0)	0 (0)	0 (0)	0 (0)	0 (0)	2 (14.3)	0 (0)	0 (0)
Other	1 (2.6)	0 (0)	0 (0)	1 (4.4)	0 (0)	0 (0)	0 (0)	0 (0)	0 (0)
**Onset Type**									
Gradual	32 (82.0)	28 (80.0)	10 (27.0)	18 (78.3)	14 (82.4)	9 (81.8)	11 (78.6)	10 (90.9)	8 (22.9)
Sudden	6 (15.4)	3 (8.6)	0 (0.0)	3 (13)	1 (5.9)	1 (9.1)	3 (21.4)	1 (9.1)	0 (0.0
Undetermined	1 (2.6)	4 (11.4)	27 (73.0)	2 (8.7)	2 (11.8)	1 (9.1)	0 (0)	0 (0)	27 (77.1)
**BMI **[n (%) ]									
<25	5 (13.8)	10 (28.6)	7 (18.9)	0 (0)	4 (23.5)	8 (72.7)	3 (21.4)	3 (27.3)	4 (11.4)
25–30	20 (51.3)	14 (40)	18 (48.7)	9 (39.1)	6 (35.3)	3 (27.3)	9 (64.3)	7 (63.6)	18 (51.4)
>30	14 (35.9)	11 (31.4)	12 (32.4)	14 (60.9)	7 (41.2)	0 (0)	2 (14.3)	1 (9.1)	13 (37.1)

^1 ^SD is standard deviation

**Table 2:** A cross-reference of systems for diagnostic assignment

		**Empiric Classification (CFS research case definition)**					
		
		**CFS**		**ISF**		**Controls**	
**LCA Category**	**LCA Class Description**	*(n = 39)*		*(n = 35)*		*(n = 37)*	
		*n*	*(%)*	*n*	*(%)*	*n*	*(%)*
		
**Controls (0–2)**	Well *(n = 33) *or Unassigned *(n = 2)*	1	(2)	0	(0)	34	(91)
**1**	Obese hypnoea *(n = 23)*	15	(38)	8	(23)	0	(0)
**3**	Obese hypnoea and stressed *(n = 17)*	5	(13)	11	(31)	1	(3)
**4**	Interoception – muscle pain *(n = 11)*	1	(3)	9	(26)	1	(3)
**5**	Interoception depression *(n = 14)*	10	(26)	4	(11)	0	(0)
**6**	Multisymptomatic, depressed, stressed *(n = 11)*	7	(18)	3	(9)	1	(3)

### Gene set development

Extracting elements that represent the abundance and activity of a specific leukocyte subset was approached by identifying discrete sets of genes that are uniquely or predominantly expressed in a given cell type [20-22]. Currently discrete gene sets offer the simplest and most immediately accessible method for analysis across microarray technological platforms. We constructed a number of gene sets a priori for CD4+ T cells, CD8+ T cells, CD19+ B cells, CD14+ monocytes and CD16+ neutrophils using data collected on Affymetrix microarrays (Affymetrix, Santa Clara, CA, USA) by Lyons et al. [23]. Of the 12,022 genes surveyed, 2,641 were differentially expressed between individual lymphocyte subsets. Of these original 2,641 distinguishing genes, 268 were present on the MWG microarrays used in the Wichita Clinical study. We further dissected these subset-specific profiles into discrete non-overlapping sets composed of genes at least 2-fold up-regulated or 2-fold down-regulated preferentially in each cell lineage. An additional gene set was defined for NK cell activity and regulatory T cell activity was estimated from the expression of the FoxP3 gene (AF277993). Individual MWG gene probes belonging to each immune gene set as well as NCBI gene annotation and PANTHER functional annotation [24,25] are listed in the supplementary data file [Additional file 1].

### Statistical analysis

The aggregate expression *G*_*a *_of each gene set *a *was computed as the average of the Ln-transformed expression Ln(*g*_*i*, *a*_) of each gene *i *across the *k *member genes in the set (Equation 1). In a first level of analysis a classical Wilcoxon non-parametric test was used to evaluate the differential expression of immune gene sets for both classification systems. As suggested by Efron and Tibshirani [15] the performance of these gene sets was also compared to that obtained with randomly populated sets of the same size. A null distribution was computed from the analysis of 1000 instances of random gene selections and 1000 random permutations of the diagnostic labels.

(1) $$G_{a}=\frac{\sum_{i=1}^{k}Ln(g_{i,a})}{k}$$

To examine the patterns of association linking immune gene sets simple linear association networks were constructed using the Pearson correlation coefficient *r*_*a*, *b *_as the metric describing similarity in the expression of gene set *a *with that of gene set *b*. Statistical significance of correlation was assessed using the *t*_*a*, *b *_statistic in Equation (2). This statistic has a Student's t-distribution with degrees of freedom *n*-2 under the null hypothesis of no correlation [26], where *n *is the number of microarray measurements. *C*_*a*, *b *_is the covariance in the expression of gene set *a *with gene set *b *and *E() *is the expected value operator or the mean.

(2) $$t_{a,b}=r_{a,b}\sqrt{\frac{\left(n-2\right)}{\left(1-r_{a,b}^{2}\right)}}$$

Where,

$$r_{a,b}=\frac{C_{a,b}}{\sqrt{C_{a,a}C_{b,b}}};C_{a,b}=E[(G_{a}-E(G_{a}))\cdot (G_{b}-E(G_{b}))]$$

A cutoff for the resulting probability p_a, b _(*t *> *t*_*a*, *b*_), above which we accept the null hypothesis, can be established in a variety of ways [27]. It should be noted however that these require specific assumptions regarding network topology such as network edge sparseness or the appearance of highly cliquish disconnected sub-networks. As such they are generally more relevant to the study of large networks. Instead we examined the dependency of the network size *S*, or the sum of the edge weights *w*_*a*, *b *_on the choice of threshold p-value (Equation 3). We compared curves obtained for NF and CFS networks, identifying threshold p-values where networks differed primarily in structure from those where they differed in both structure and size.

(3) $$\begin{array}{l} S=\sum_{a=1}^{M-1}\sum_{b>a}^{M}w_{a,b}\\ \begin{array}{ccc} \text{where} & w_{a,b}=r_{a,b} & {\text{if p}}_{\text{a},\text{b}}\leq {\text{p}}_{\text{threshold}}\\ & w_{a,b}=0 & {\text{if p}}_{\text{a},\text{b}}>{\text{p}}_{\text{threshold}} \end{array} \end{array}$$

## Results

### Alignment of empiric and LCA classifications

A cross tabulation of the empiric classification and LCA classification is presented in Table 2. There was good alignment of non-fatigued subjects with 90% of empiric NF controls residing in LCA classes 0 (Well) and 2 (Unassigned). Together LCA classes 1 (40%) and 5 (26%) contained two thirds of the subjects assigned to the empiric CFS class. However ISF subjects were distributed almost equally across LCA classes 1 (23%), 3 (31%), and 4 (26%). Conversely most LCA class 3 and 4 subjects were identified as ISF and most subjects in LCA classes 1, 5 and 6 were assigned an empiric CFS classification.

### Differential expression of a priori defined immune cell gene sets

In a first level of analysis the differential expression of immune gene sets across disease phenotypes and control groups for both classification systems was evaluated. Results in Table 3 show that the median expression of the CD19+ B cell up-regulated gene set was significantly lower in CFS (p = 0.01) and ISF (p = 0.05) subjects when compared to the NF group. Expression of this gene set was also significantly repressed in LCA class 3 (p = 0.04) and marginally so in LCA class 5 (p = 0.09) when compared to control subjects in LCA classes 0 and 2. Recall that 11 of 17 cases in LCA class 3 were also designated ISF. Similarly 10 of the 14 LCA class 5 cases were designated CFS. NK gene set expression was marginally increased in the CFS group (p = 0.07). Though not significant the null probability for NK cell expression was lowest among the LCA classes for LCA-3 (p = 0.11). Finally expression of the T regulatory set (FoxP3) was marginally repressed in LCA class 1 (p = 0.09) which contained 40% of the CFS subjects though no significant difference was found for the larger CFS group (p = 0.31).

**Table 3:** Changes in median expression and corresponding null probability values () for pair-wise comparison of disease classes and the non-fatigued control group under both classification systems

			3-Class (NF Controls)		7-Class (Controls = LCA-0 U LCA-2)				
	
**Cell Type**	**Expression Level +/-**	**Number of genes**	ISF	CFS	LCA-1	LCA-3	LCA-4	LCA-5	LCA-6
	
CD8 T cells	Up-regulated	5	0.06 (0.43)	0.01 (0.75)	0.06 (0.52)	0.00 (0.64)	0.04 (0.24)	0.01 (0.80)	0.23 (0.27)
	Down-regulated								
CD14 Monocytes	Up-regulated	78	0.04 (0.29)	0.05 (0.83)	0.02 (0.62)	0.11 (0.15)	0.09 (0.42)	0.02 (0.94)	0.05 (0.33)
	Down-regulated								
CD16 Neutrophils	Up-regulated								
	Down-regulated	185	0.02 (0.20)	0.02 (0.25)	0.02 (0.29)	0.00 (0.65)	0.03 (0.25)	0.02 (0.50)	0.04 (0.30)
CD19 B cells	Up-regulated	6	-0.17 (0.05)	-0.28 (0.01)	-0.27 (0.29)	-0.31 (0.04)	-0.12 (0.13)	-0.25 (0.09)	-0.17 (0.14)
	Down-regulated	2	-0.22 (0.33)	-0.22 (0.29)	-0.18 (0.19)	0.07 (0.71)	0.10 (0.76)	-0.08 (0.78)	-0.08 (0.16)
CD4/8/25 T reg cells	NA	1	-0.23 (0.19)	-0.23 (0.31)	-0.35 (0.09)	0.19 (0.37)	-0.25 (0.11)	-0.17 (0.32)	0.28 (0.40)
NK cells	NA	4	0.09 (0.92)	0.30 (0.07)	0.19 (0.20)	0.32 (0.11)	0.12 (0.68)	0.12 (0.45)	-0.01 (0.57)

**Figure 1:**
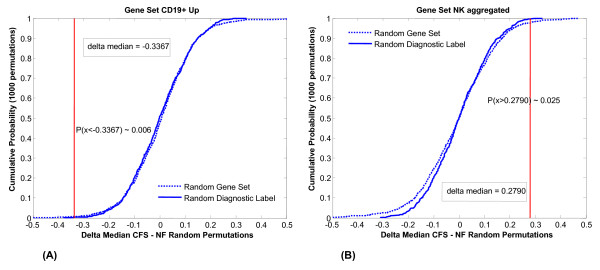
Cumulative probability plot of Δ differential expression of CFS versus NF for random gene sets similar in size to the CD19+ B cell up-regulated gene set and the NK cell gene set.

**Figure 2:**
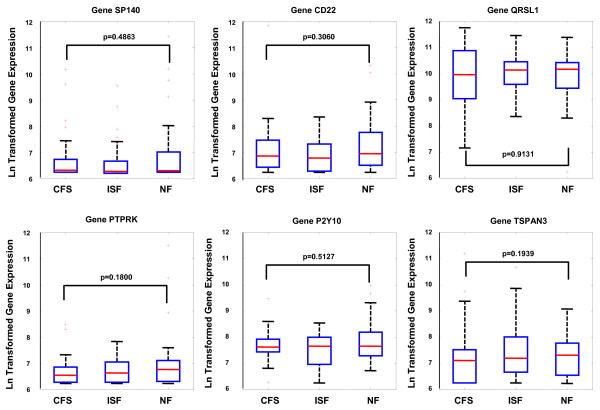
**Box and whisker plot for the expression of each gene in the CD19+ up-regulated gene set in each of the 3 empiric illness classes.** Boxes indicate the lower quartile, median and upper quartile values. Whiskers are located at extreme values within 1.5 times the inter-quartile range from the ends of each box. Outliers are displayed with a red '+'. Each plot is annotated with the null probability for the difference in median expression between the NF and CFS subject groups.

**Figure 3:**
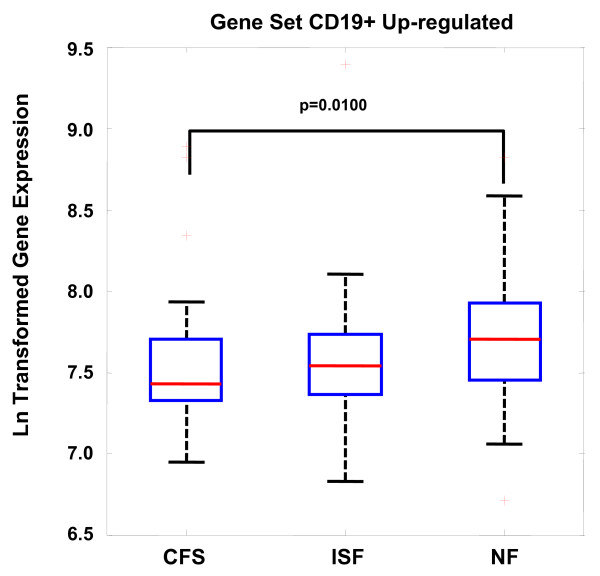
**Box and whisker plot for the expression of the CD19+ up-regulated gene set in each of the 3 empiric illness classes.** Boxes indicate the lower quartile, median and upper quartile values. Whiskers are located at extreme values within 1.5 times the inter-quartile range from the ends of each box. Outliers are displayed with a red '+'. The plot is annotated with the null probability for the difference in median expression between the NF and CFS subject groups.

The performance of these gene sets was compared to that obtained with randomly populated sets of the same size as well as by random classification assignment of each subject. Null distribution results indicated that both the CD19+ B cell (up-regulated) and NK cell gene sets performed significantly better than random sets of equivalent size in discriminating CFS from NF (p < 0.05) (Figure 1). Performance of the T regulatory gene set (FoxP3) was marginal at best (p~0.15) in terms of uniqueness in differential expression. In addition a detailed analysis of individual genes in the CD19+ up-regulated set indicated that no single gene was differentially expressed even though the parent set was expressed at the p = 0.01 level. This reaffirms that high levels of measurement noise can be effectively managed by aggregating genes into biologically relevant sets. Details of this analysis are listed in Table 4 and illustrated graphically in Figure 2 and Figure 3.

**Table 4:** Changes in median expression and corresponding null probability values () for pair-wise comparison of disease classes and the non-fatigued control group for each individual gene in the CD19+ B cell Up-regulated gene set

	3-Class (NF Controls)		7-Class (Controls = LCA-0 U LCA-2)				
	
**Gene**	ISF	CFS	LCA-1	LCA-3	LCA-4	LCA-5	LCA-6
	
SP140	-0.03 (0.60)	0.02 (0.49)	-0.07 (0.43)	0.10 (0.60)	0.01 (0.72)	-0.08 (0.29)	0.07 (0.92)
CD22	-0.16 (0.10)	-0.09 (0.31)	-0.14 (0.17)	-0.58 (0.02)	-0.30 (0.04)	0.02 (0.35)	0.05 (0.70)
QRSL1	-0.03 (0.95)	-0.21 (0.91)	0.29 (0.28)	-0.10 (0.70)	0.23 (0.50)	-0.16 (0.89)	-0.49 (0.42)
PTPRK	-0.12 (0.55)	-0.21 (0.18)	-0.12 (0.76)	-0.07 (0.82)	-0.22 (0.68)	-0.20 (0.17)	-0.07 (0.64)
P2RY10	0.01 (0.21)	-0.02 (0.51)	0.08 (0.99)	-0.08 (0.06)	0.28 (0.82)	0.11 (0.94)	0.20 (0.33)
TSPAN3	-0.10 (0.54)	-0.20 (0.19)	-0.24 (0.39)	-0.17 (0.83)	0.13 (0.38)	-0.08 (0.83)	-0.02 (0.82)

### Emergence of characteristic patterns of association between immune gene sets in CFS

**Figure 4:**
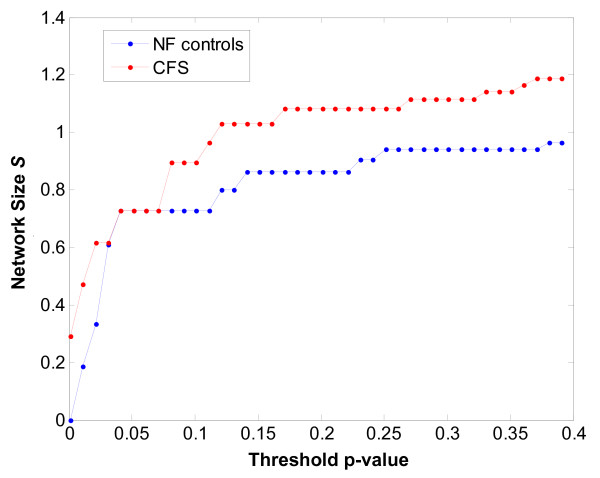
Network size *S *defined as the sum of all network edge weights (Equation 3) and plotted as a function of cutoff p-value for the empiric NF and CFS classes.

Conventional analysis of microarray data remains focused on the detection of differentially expressed genes or gene sets. However it is important to realize that genes expressed at similar levels across patient groups may still play an important role in the disease process. To examine the patterns of association linking immune gene sets simple linear correlation networks were constructed. Results in Figure 4 show network size for the empiric NF and CFS classes as a function of cutoff p-value for edge weight significance. Both NF and CFS networks were identical in overall size at a cutoff p-value of 0.05. Changes at this level of edge weight significance consisted therefore of a re-organization of edges only. The curves in Figure 4 diverge at p~0.10 and maintain a similar offset form one another as p-value increases. As a result comparisons of network topology conducted at the p < 0.10 level included edge re-assignment as well as the addition of new edges to the CFS network. Topologies emerging at both p < 0.05 and p < 0.10 thresholds were examined as they contain complementary information. Detailed results of pair-wise correlation between gene sets may be found in Table 5 for the empiric classification system and Tables 6 and 7 for the LCA classification system.

**Table 5:** Detailed results of gene set correlation *r*_*a*, *b*_(null probability p_a, b_) for empiric classes.

**CFS**	CD16+ Down	CD14+ Up	CD19+ Up	CD19+ Down	CD8+ Up	T reg	NK cell
CD16+ Down		**0.43 (0.01)**	0.13 (0.42)	0.14 (0.39)	**0.33 (0.04)**	0.27 (0.10)	-0.25 (0.12)
CD14+ Up			**0.54 (0.00)**	**0.29 (0.07)**	-0.12 (0.46)	0.18 (0.26)	-0.08 (0.61)
CD19+ Up				0.16 (0.33)	**-0.38 (0.02)**	0.12 (0.46)	0.15 (0.36)
CD19+ Down					0.15 (0.37)	-0.23 (0.16)	-0.04 (0.79)
CD8+ Up						0.00 (1.00)	**-0.29 (0.08)**
T reg							0.02 (0.88)
NK cell							

							

**ISF**	CD16+ Down	CD14+ Up	CD19+ Up	CD19+ Down	CD8+ Up	T reg	NK cell

CD16+ Down		0.13 (0.46)	0.11 (0.51)	-0.13 (0.47)	-0.16 (0.37)	0.19 (0.27)	0.09 (0.62)
CD14+ Up			-0.24 (0.17)	0.09 (0.62)	-0.26 (0.13)	-0.11 (0.52)	**0.49 (0.00)**
CD19+ Up				-0.08 (0.64)	0.02 (0.91)	0.26 (0.13)	-0.03 (0.88)
CD19+ Down					0.22 (0.21)	0.00 (0.99)	**-0.30 (0.08)**
CD8+ Up						0.17 (0.33)	-0.26 (0.13)
T reg							-0.04 (0.80)
NK cell							

							

**NF**	CD16+ Down	CD14+ Up	CD19+ Up	CD19+ Down	CD8+ Up	T reg	NK cell

CD16+ Down		-0.10 (0.55)	0.11 (0.53)	0.08 (0.64)	-0.27 (0.11)	0.15 (0.38)	-0.01 (0.96)
CD14+ Up			0.12 (0.49)	-0.06 (0.74)	**-0.38 (0.02)**	-0.10 (0.54)	0.02 (0.91)
CD19+ Up				**0.43 (0.01)**	-0.02 (0.90)	**0.37 (0.02)**	0.25 (0.13)
CD19+ Down					0.05 (0.79)	**0.37 (0.02)**	**0.35 (0.04)**
CD8+ Up						-0.10 (0.58)	0.20 (0.24)
T reg							0.20 (0.23)
NK cell							

**Table 6:** Detailed results of gene set correlation *r*_*a*, *b*_(null probability p_a, b_) for LCA-0/2, 1, 3 classes.

**LCA-0/2**	CD16+ Down	CD14+ Up	CD19+ Up	CD19+ Down	CD8+ Up	T reg	NK cell
CD16+ Down		-0.17 (0.33)	0.15 (0.39)	0.09 (0.62)	-0.19 (0.27)	0.09 (0.62)	-0.10 (0.56)
CD14+ Up			0.14 (0.41)	-0.03 (0.88)	**-0.30 (0.08)**	-0.11 (0.52)	0.00 (0.98)
CD19+ Up				**0.43 (0.01)**	-0.04 (0.82)	**0.40 (0.02)**	0.19 (0.28)
CD19+ Down					-0.01 (0.93)	**0.39 (0.02)**	**0.30 (0.08)**
CD8+ Up						-0.05 (0.77)	0.06 (0.73)
T reg							0.04 (0.82)
NK cell							

							

**LCA-1**	CD16+ Down	CD14+ Up	CD19+ Up	CD19+ Down	CD8+ Up	T reg	NK cell

CD16+ Down		**0.55 (0.01)**	-0.03 (0.91)	0.16 (0.47)	0.28 (0.20)	0.16 (0.46)	-0.25 (0.25)
CD14+ Up			0.25 (0.26)	0.20 (0.37)	-0.03 (0.90)	**0.36 (0.10)**	-0.04 (0.84)
CD19+ Up				-0.14 (0.53)	-0.21 (0.34)	0.13 (0.55)	0.06 (0.77)
CD19+ Down					0.33 (0.13)	-0.19 (0.38)	**-0.41 (0.05)**
CD8+ Up						0.09 (0.69)	-0.32 (0.14)
T reg							-0.02 (0.91)
NK cell							

							

**LCA-3**	CD16+ Down	CD14+ Up	CD19+ Up	CD19+ Down	CD8+ Up	T reg	NK cell

CD16+ Down		-0.13 (0.61)	0.07 (0.80)	-0.18 (0.49)	-0.20 (0.45)	0.35 (0.16)	-0.31 (0.22)
CD14+ Up			-0.31 (0.23)	0.15 (0.57)	-0.36 (0.16)	**-0.45 (0.07)**	0.15 (0.56)
CD19+ Up				0.37 (0.14)	-0.06 (0.82)	0.29 (0.26)	-0.07 (0.78)
CD19+ Down					0.20 (0.44)	0.15 (0.56)	0.18 (0.48)
CD8+ Up						0.21 (0.42)	-0.05 (0.84)
T reg							0.19 (0.47)
NK cell							

**Table 7:** Detailed results of gene set correlation *r*_*a*, *b*_(null probability p_a, b_) for LCA-4, 5, 6 classes.

**LCA-4**	CD16+ Down	CD14+ Up	CD19+ Up	CD19+ Down	CD8+ Up	T reg	NK cell
CD16+ Down		0.41 (0.21)	-0.02 (0.96)	-0.09 (0.80)	-0.14 (0.68)	0.00 (0.99)	0.14 (0.67)
CD14+ Up			-0.05 (0.88)	-0.13 (0.71)	-0.12 (0.73)	-0.01 (0.98)	-0.28 (0.41)
CD19+ Up				-0.21 (0.53)	0.09 (0.78)	0.28 (0.40)	0.13 (0.71)
CD19+ Down					0.44 (0.18)	0.05 (0.89)	-0.48 (0.13)
CD8+ Up						0.19 (0.57)	-0.04 (0.91)
T reg							-0.11 (0.75)
NK cell							

							

**LCA-5**	CD16+ Down	CD14+ Up	CD19+ Up	CD19+ Down	CD8+ Up	T reg	NK cell

CD16+ Down		0.44 (0.12)	0.35 (0.22)	0.05 (0.87)	0.32 (0.27)	0.17 (0.56)	0.04 (0.90)
CD14+ Up			**0.86 (0.00)**	0.23 (0.44)	-0.40 (0.16)	0.35 (0.21)	0.40 (0.16)
CD19+ Up				0.15 (0.60)	**-0.48 (0.08)**	0.35 (0.22)	0.36 (0.20)
CD19+ Down					-0.16 (0.58)	0.16 (0.60)	-0.16 (0.59)
CD8+ Up						-0.22 (0.45)	-0.12 (0.69)
T reg							0.07 (0.82)
NK cell							

							

**LCA-6**	CD16+ Down	CD14+ Up	CD19+ Up	CD19+ Down	CD8+ Up	T reg	NK cell

CD16+ Down		0.34 (0.30)	-0.02 (0.95)	0.20 (0.55)	-0.15 (0.65)	0.45 (0.16)	0.23 (0.50)
CD14+ Up			0.10 (0.76)	0.57 (0.07)	-0.14 (0.69)	-0.40 (0.22)	0.20 (0.56)
CD19+ Up				0.35 (0.30)	-0.33 (0.32)	-0.43 (0.18)	0.31 (0.35)
CD19+ Down					0.15 (0.67)	-0.31 (0.36)	0.32 (0.33)
CD8+ Up						0.11 (0.74)	-0.46 (0.16)
T reg							0.07 (0.84)
NK cell							

^1 ^SD is standard deviation

**Figure 5:**
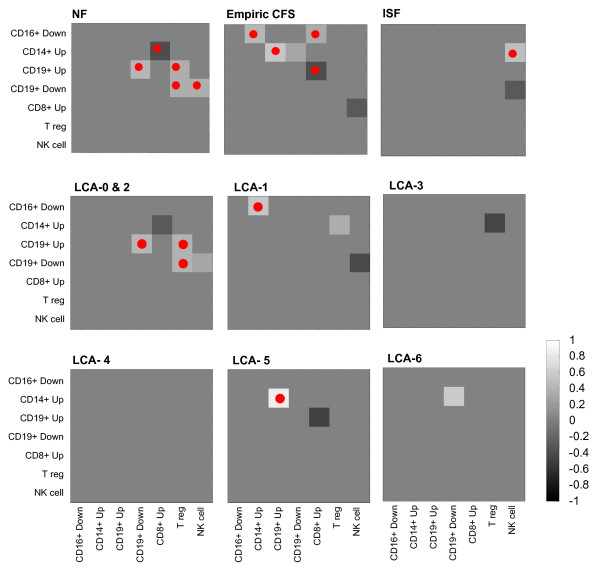
Heat maps of gene set co-expression expressed as linear correlation coefficient *r*_*a*, *b *_at cutoff significance p_a,b_<0.10 (□) and at cutoff significance p_a,b _<0.05 (●) for empiric classes for non-fatigued (NF) controls, insufficient fatigue symptoms (ISF) and CFS as well as for LCA control classes (LCA-0, 2) and for all LCA disease classes.

Heat maps depicting the edge weights *r*_*a*, *b *_linking gene sets are presented in Figure 5 for both empiric and LCA classes. The LCA control classes 0 and 2 exhibited a pattern of gene set co-expression at the p < 0.10 identical to that of the empiric NF group though in the latter this pattern was also retained at the p < 0.05 level. In both the NF and LCA-0/LCA-2 networks CD 19+ B cell up-regulated and down-regulated gene sets correlated tightly behaving as one set (*r *= 0.43, p = 0.008). T regulatory and NK cell gene sets both supported significant positive interaction with one or both of the CD19+ B cell sets. In addition CD8+ T cell activity and CD14+ monocyte activity were significantly antagonistic. In contrast the network obtained for the CFS subjects displayed a shift in interactions towards the upper left hand corner of the heat map. Indeed significant interactions appeared linking the expression of the CD14+ monocyte gene set with that of the B cell (CD19+ up-regulated) and the CD16+ neutrophil gene sets. The neutrophil set also shared significant co-expression with the CD8+ T cell gene set (p < 0.05) in CFS. Interestingly interactions with the neutrophil gene set were completely absent in NF even at the p < 0.10 level. Also apparent in CFS was the emergence of a significant negative correlation between the expression of CD8+ and CD19+ up-regulated gene sets (p = 0.02). Moreover CD19+ B cells appeared altered with up and down-regulated sets no longer maintaining a strong direct correlation in ISF or CFS. Interaction with NK cell gene set expression was also a distinguishing feature in particular for ISF. Instead of appearing as a transitional state between NF and CFS, ISF exhibited a distinct co-expression pattern characterized by a significant interaction of NK cell and monocyte gene sets (p < 0.05). Contrary to NF, the NK and CD19+ down-regulated gene sets correlated negatively (p < 0.10) in ISF.

In much the same way as the ISF group, several of the LCA groups were characterized by a lack of coordinated activity between immune gene sets. Indeed no significant correlations existed for LCA-4 even at the p < 0.10 level. This was also true of LCA-3 and LCA-6 classes at the p < 0.05 level. Though also quite sparse, heat maps for LCA-1 and LCA-5 each recovered specific features of the CFS and ISF heat maps. LCA-1 demonstrated a significant positive correlation (p < 0.05) between CD14+ monocyte and CD16+ neutrophil sets, a CFS feature. At the p < 0.10 level the same heat map showed a negative correlation between the NK and CD19+ B cell down-regulated set, an ISF feature. Unique to LCA-1 was a positive correlation between T reg (FoxP3) and CD14+ monocyte gene set expression (p < 0.10), a trait not identified in CFS and actually reversed in LCA-3. Similarly the heat map for LCA-5 contained 2 features specific to the CFS group, namely a strong positive correlation linking CD19+ B cell with CD14+ monocyte up-regulated sets (p < 0.05) along with a strong negative correlation linking the former with CD8+ gene set expression. These 2 features were not shared with the LCA-1 group. These results reaffirmed the strong links between the CFS subject group and LCA classes 1 and 5 in addition to suggesting that immune set co-expression might offer insight into the distinct nature of these apparent subclasses of CFS.

## Discussion

In this work we dissected PBMC gene expression profiles into components that were preferentially expressed in several isolated lymphocyte subpopulations. We also used 2 systems to stratify subjects into illness groups. The LCA class structure was inferred directly from a comprehensive set of clinical and biological indicators. All indicators were equally weighted and contrary to common practice no subset was assigned greater relevance a priori. In contrast the empiric classification which was based on a consensus of opinions from expert clinicians. Results confirm strong links between both systems with the LCA classification providing additional insight into potential subclasses of CFS. The commonalities between these classification systems are readily observed in the patterns of gene set co-expression. Indeed the empiric CFS group seems to present an aggregation of the gene set co-expression patterns observed in LCA classes 1 and 5. However, the differential expression of gene sets only achieves statistical significance in the case of the coarser empiric classes with the larger group sizes providing better noise reduction. Specifically in the empiric CFS class we found a significant decrease in the median expression for a set of 6 genes preferentially up-regulated in isolated CD19+ B cells compared to non-fatigued controls. Expression of this CD19+ B cell up-regulated gene set also discriminated ISF from controls at 0.05 confidence level. In a recent study of CFS occurrence both in the presence and absence of viral infection Racciati et al. [8] found no significant differences in CD19+ cell abundance. Robertson et al. [7] recently reported significantly higher *abundance *of CD20+/CD5+ B cells, a subset associated with the production of auto-antibodies, in patients with depression. These findings together with our observations of depressed CD19+ gene expression and altered association between up and down-regulated B cell functions would suggest that the function of these cells might be compromised in CFS subjects. Cole et al. [28] reported a selective reduction of mature B lymphocyte function in subjects who experienced chronic high levels of social isolation including suppression of several transcription factors involved B cell differentiation such as Ikaros/ZNF1A1. Genes encoding for members of the zinc finger protein family were also identified in previous work by this group as prominent contributors to the CFS symptom space [9]. A closer look at the 6 genes that constitute the CD19+ up-regulated set showed that the PTPRK and TSPAN3 genes, both associated with immune cell adhesion and development, were the most suppressed. Down-regulation of PTPRK, a TGF-β target gene, is known to be down-regulated by the Epstein-Barr virus (EBV) [29], an infectious agent known to trigger CFS [30,31]. Down-regulation of TGF-β has been reported in CFS by Tomoda et al. [32].

NK cell activity is suppressed in CFS [33] and this decreased cytotoxity has been associated with reduced intracellular perforin [34]. In this work we observe an increased expression of the NK cell gene set. Of the 4 genes used to capture NK cell function the expression of NKG2A/C (NM 002260) was most increased. The binding of NKG2A to its natural ligand, human non-classic class I leukocyte antigen (HLA) E, is known to induce its immunoreceptor tyrosine-based inhibition motif (ITIM) and suppress cytotoxic cell effector activity [35]. Moreover NKG2A is also known to be co-expressed on activated Th2 but not Th1 lymphocytes [36]. A bias towards Th2-type immune response in CFS patients has also been suggested on the basis of intracellular T cell cytokine profiles by Skowera et al. [37]. Interestingly this also aligns with altered expression of the PTPRK gene mentioned above as Asano et al. [38] report impaired Th1 function with PTPRK deletion in rats. Therefore our observations supported findings of increased suppression of cytotoxic activity in CFS and hinted at increased Th2 activity though the latter were not specifically addressed in this analysis.

Neutrophils for their part are only found at trace and contaminating amounts in most PBMC preparations [39] so it is interesting to note that the neutrophil gene set arose as a core element in the emergence of coordinated immune activity. In particular the CD16+ neutrophil gene set and the CD14+ monocyte gene set shared significant co-expression. Not only do these arise from the same hematopoietic CD34+ progenitor cell [40] but since the immune community is highly integrated the presence or absence of neutrophils will also be mirrored in the state of the remaining cell population. The CD14+ monocyte set also shared significant co-expression with the CD19+ B cell gene sets. Together this neutrophil-monocyte-B cell immune interaction triad is highly consistent with a model of chronic inflammation proposed by Lefkowitz and Lefkowitz [41]. According to this model once an event initiates inflammation, neutrophils are among the first cells to arrive at the site. They degranulate releasing MPO into the microenvironment which together with iMPO binds to macrophage MMR receptor and induces release of TNF-α. The latter functions in an autocrine manner and along with iMPO initiates a cytokine cascade (IL-1, IL-6, IL-8, GM-CSF). IL-8 attracts more neutrophils and together with GM-CSF causes these to once again degranulate. With the corresponding release of additional MPO, the cycle starts once again. The TNF-α initiated cascade induces IL-6 which is used by B cells for maximum antibody secretion usually IgM. In addition to the present analysis, a preliminary examination of cytokine data collected in the Wichita study pointed to an increase in TNF-α in CFS subjects (data not shown) as documented previously by Moss et al. [42].

In addition to this core network, we also observed that CD8+ T cell set expression correlated negatively with that of the NK and CD19+ up-regulated B cell sets. In one possible mechanism linking these three cell types, IgG antibodies binding to GD3 on the surface of CD4+ and CD8+ T cells could elicit signals for proliferation of these subsets and expression of the IL-2 receptor CD25. NK cells have been shown to selectively inhibit this antibody-mediated proliferation of CD8+ T cells by Claus et al. [43] perhaps through down-regulation of autologous mixed lymphocyte reaction (MLR). This basic analysis of immune gene set co-expression points therefore to the existence of immune signaling processes in CFS that adhere to at least one known mechanism of chronic inflammation and support possible antibody-mediated NK cell modulation of T cell activity. Furthermore association networks constructed for LCA classes 1 and 5 suggested that B cell involvement in these processes may serve as factor for discriminating between distinct subsets of CFS subjects.

Although several very plausible immune response mechanisms were recovered by this analysis it must be emphasized that the use of discrete gene sets has several limitations. In particular it becomes increasingly difficult to identify genes that are exclusively or even predominantly expressed in specific cell lineages when these share many commonalities of function and goal. This issue was reflected in by the small size of the gene sets identified in this work from lymphocyte subset expression profiles. An approach that promises to be more robust and more revealing still involves the direct use of the genome-wide expression for these cell populations. This remains an active area of research [44]. However, even this simple analysis points to dramatic differences in immune network topology and cell signaling in CFS and we expect these differences to be largely conserved in more elaborate analyses. Furthermore the methodology outlined and issues raised in this work demonstrate the importance of developing approaches that effectively integrate flow cytometry with cytokine and gene expression profiling. In particular it underscores the importance of looking beyond differential expression of individual components towards changes in their patterns of coordinated activity and formally recognizing the network properties of the immune system.

## List of abbreviations

CD: cluster of differentiation; CDC: Centers for disease Control and Prevention; cDNA: complementary DNA; CFS: Chronic Fatigue Syndrome; DPP-IV: dipeptidyl peptidase-4; EBV: Epstein-Barr virus; FoxP3: forkhead box P3 gene; GD3: ganglioside D3; GM-CSF: granulocyte macrophage colony-stimulating factor; HCMV: human cytomegalovirus; Ig: immunoglobulin; iMPO: inactive myeloperoxidase; IL: interleukin; ISF: insufficient symptoms of fatigue; MFI: Multidimensional Fatigue Inventory; MLR: mixed lymphocyte reaction; MMR: macrophage; mannose receptor; NF: non-fatigued; NK: natural killer cell; NKG2A/C: killer cell lectin-like receptor subfamily C, member 1/2 or KLRC1/C2; LCA: latent class analysis; MPO: myeloperoxidase; PANTHER: Protein ANalysis THrough Evolutionary Relationships; PBMC: peripheral blood mononuclear cell; PTPRK: protein tyrosine phosphatase, receptor type, K; SF-36: Short Form 36; TGF: transforming growth factor; TNF: tumor necrosis factor; Th: T helper cell; TSPAN3: tetraspanin 3.

## Competing interests

The authors declare that they have no competing interests.

## Authors' contributions

ALA carried out statistical analysis of gene expression profiles, gene set design and significance analysis and helped draft the manuscript. CB participated in the design of the study and assisted with the statistical analysis and helped draft the manuscript. SDV helped conceive of the study, its design and coordination and helped to draft the manuscript. GB conceived of the study, its design and coordination, supervised the analysis and drafted the manuscript. All authors read and approved the final manuscript.

## Supplementary Material

Additional File 1 Supplementary_data_Panther_Gene_List_Annotation. Additional data file 1 is a set of tables listing the genes used in each gene set along with the functional annotation available in the PANTHER classification system. This file also contains the median values and standard deviations within each illness class of the Ln-transformed gene expression for each gene. Click here for file

## References

[B1] Reynolds KJ, Vernon SD, Bouchery E, Reeves WC The economic impact of chronic fatigue syndrome. Cost Eff Resour Alloc.

[B2] Klimas N, Salvato F, Morgan R, Fletcher MA (1990). Immunologic abnormalities in chronic fatigue syndrome. J Clin Microbiol.

[B3] Straus SE, Fritz S, Dale JK, Gould B, Strober W (1993). Lymphocyte phenotyping and function in chronic fatigue syndrome. J of Clin Immunol.

[B4] Tirelli U, Bernardi D, Improta S, Pinto A (1996). Immunologic abnormalities in chronic fatigue syndrome. J Chronic Fatigue Syndrome.

[B5] Yan S, Marguet D, Dobers J, Reutter W, Fan H (2003). Deficiency of CD26 results in a change of cytokine and immunoglobulin secretion after stimulation by pokeweed mitogen. Eur J Immunol.

[B6] Boonacker E, Van Noorden CJ (2003). The multifunctional or moonlighting protein CD26/DPPIV. Eur J Cell Biol.

[B7] Robertson MJ, Schacterle RS, Mackin GA, Wilson SN, Bloomingdale KL, Ritz J, Komaroff AL (2005). Lymphocyte subset differences in patients with chronic fatigue syndrome, multiple sclerosis and major depression. Clin Exp Immunol.

[B8] Racciatti D, Dalessandro M, Delle Donne L, Falasca K, Zingariello P, Paganelli R, Pizzigallo E, Vecchiet J (2004). Study of immune alteration in patients with chronic fatigue syndrome with different etiologies. J Immunopath Pharm.

[B9] Broderick G, Craddock RC, Whistler T, Taylor R, Klimas N, Unger ER (2006). Identifying illness parameters in fatiguing syndromes using classical projection methods. Pharmacogenomics.

[B10] Carmel L, Efroni S, White PD, Aslakson E, Vollmer-Conna U, Rajeevan MS (2006). Gene expression profile of empirically delineated classes of unexplained chronic fatigue. Pharmacogenomics.

[B11] Whistler T, Jones JF, Unger ER, Vernon SD Exercise responsive genes measured in peripheral blood of women with chronic fatigue syndrome and matched control subjects. BMC Physiol.

[B12] Siegel S, Antoni M, Fletcher M, Maher K, Segota M, Klimas N (2006). Impaired natural immunity, cognitive dysfunction, and physical symptoms in patients with chronic fatigue syndrome: preliminary evidence for a subgroup?. J Psychosom Res.

[B13] Segal E, Friedman N, Koller D, Regev A (2004). A module map showing conditional activity of expression modules in cancer. Nat Genet.

[B14] Dinu I, Potter JD, Mueller T, Liu Q, Adewale AJ, Jhangri GS, Einecke G, Famulski KS, Halloran P, Yasui Y Improving gene set analysis of microarray data by SAM-GS. BMC Bioinformatics.

[B15] Efron B, Tibshirani R (2007). On testing the significance of sets of genes. Ann Appl Statist.

[B16] Vernon SD, Reeves WC (2006). The challenge of integrating disparate high-content data: epidemiologic, clinical, and laboratory data collected during an in-hospital study of chronic fatigue syndrome. Pharmacogenomics.

[B17] Reeves WC, Wagner D, Nisenbaum R, Jones JF, Gurbaxani B, Solomon L, Papanicolaou DA, Unger ER, Vernon SD, Heim C (2005). Chronic fatigue syndrome – A clinically empirical approach to its definition and study. BMC Medicine.

[B18] Wagner D, Nisenbaum R, Heim C, Jones JF, Unger ER, Reeves WC (2005). Psychometric properties of the CDC Symptom Inventory for assessment of chronic fatigue syndrome. Popul Health Metr.

[B19] Vollmer-Conna U, Aslakson E, White PD (2006). An empirical delineation of the heterogeneity of chronic unexplained fatigue in women. Pharmacogenomics.

[B20] Einecke G, Broderick G, Sis B, Halloran PF (2007). Early loss of renal transcripts in kidney allografts: relationship to morphologic changes and alloimmune effector mechanisms. Am J Transplant.

[B21] Famulski KS, Broderick G, Einecke G, Hay K, Cruz J, Sis B, Mengel M, Halloran PF (2007). Transcriptome analysis reveals heterogeneity in the injury response of kidney transplants. Am J Transplant.

[B22] Mueller TF, Einecke G, Reeve J, Sis B, Mengel M, Jhangri GS, Bunnag S, Cruz J, Wishart D, Meng C, Broderick G, Kaplan B, Halloran PF (2007). Microarray Analysis of Rejection in Human Kidney Transplants Using Pathogenesis-based Transcripts Sets. Am J Transplant.

[B23] Lyons PA, Koukoulaki M, Hatton A, Doggett K, Woffendin HB, Chaudhry AN, Smith KG (2007). Microarray analysis of human leucocyte subsets: the advantages of positive selection and rapid purification. BMC Genomics.

[B24] Thomas PD, Kejariwal A, Campbell MJ, Mi H, Diemer K, Guo N, Ladunga I, Ulitsky-Lazareva B, Muruganujan A, Rabkin S, Vandergriff JA, Doremieux O (2003). PANTHER: a browsable database of gene products organized by biological function, using curated protein family and subfamily classification. Nucleic Acids Res.

[B25] PANTHER Classification System. http://www.pantherdb.org.

[B26] Bernstein S, Bernstein R (1999). Schaum's Outline of Theory and Problems of Elements of Statistics II.

[B27] Elo LL, Järvenpää H, Orešiè M, Lahesmaa R, Aittokallio T (2007). Systematic construction of gene coexpression networks with applications to human T helper cell differentiation process. Bioinformatics.

[B28] Cole SW, Hawkley LC, Arevalo JM, Sung CY, Rose RM, Cacioppo JT (2007). Social regulation of gene expression in human leukocytes. Genome Biology.

[B29] Flavell JR, Baumforth KR, Wood VH, Davies GL, Wei W, Reynolds GM, Morgan S, Boyce A, Kelly GL, Young LS, Murray PG (2008). Downregulation of the TGF-Beta target gene, PTPRK, by the epstein-barr virus encoded EBNA1 contributes to the growth and survival of Hodgkin's lymphoma cells. Blood.

[B30] Lerner AM, Beqaj SH, Deeter RG, Fitzgerald JT (2004). IgM serum antibodies to Epstein-Barr virus are uniquely present in a subset of patients with the chronic fatigue syndrome. In Vivo.

[B31] Hickie I, Davenport T, Wakefield D, Vollmer-Conna U, Cameron B, Vernon SD, Reeves WC, Lloyd A, Dubbo Infection Outcomes Study Group Post-infective and chronic fatigue syndromes precipitated by viral and non-viral pathogens: prospective cohort study. BMJ.

[B32] Tomoda A, Joudoi T, Rabab el-M, Matsumoto T, Park TH, Miike T (2005). Cytokine production and modulation: comparison of patients with chronic fatigue syndrome and normal controls. Psychiatry Res.

[B33] Fletcher MA, Maher K, Klimas NG (2002). Natural killer cell function in chronic fatigue syndrome. Clin Applied Immunol Rev.

[B34] Maher KJ, Klimas NG, Fletcher MA (2005). Chronic fatigue syndrome is associated with diminished intracellular perforin. Clin Exp Immunol.

[B35] Kabat J, Borrego F, Brooks A, Coligan JE (2002). Role that each NKG2A immunoreceptor tyrosine-based inhibitory motif plays in mediating the human CD94/NKG2A inhibitory signal. J Immunol.

[B36] Freishtat RJ, Mitchell LW, Ghimbovschi SD, Meyers SB, Hoffman EP (2005). NKG2A and CD56 are coexpressed on activated TH2 but not TH1 lymphocytes. Hum Immunol.

[B37] Skowera A, Cleare A, Blair D, Bevis L, Wessely SC, Peakman M (2004). High levels of type 2 cytokine-producing cells in chronic fatigue syndrome. Clin Exp Immunol.

[B38] Asano A, Tsubomatsu K, Jung CG, Sasaki N, Agui T (2007). A deletion mutation of the protein tyrosine phosphatase kappa (Ptprk) gene is responsible for T-helper immunodeficiency (thid) in the LEC rat. Mamm Genome.

[B39] Jison ML, Munson PJ, Barb JJ, Suffredini AF, Talwar S, Logun C, Raghavachari N, Beigel JH, Shelhamer JH, Danner RL, Gladwin MT Blood mononuclear cell gene expression profiles characterize the oxidant, hemolytic, and inflammatory stress of sickle cell disease. Blood.

[B40] Ferrari F, Bortoluzzi S, Coppe A, Basso D, Bicciato S, Zini R, Gemelli C, Danieli GA, Ferrari S Genomic expression during human myelopoiesis. BMC Genomics.

[B41] Lefkowitz DL, Lefkowitz SS (2001). Macrophage-neutrophil interaction: A paradigm for chronic inflammation revisited. Immunol Cell Biol.

[B42] Moss RB, Mercandetti A, Vojdani A (1999). TNF-alpha and chronic fatigue syndrome. J Clin Immunol.

[B43] Claus C, Schlaak J, Dithnayer M, Meyer zum K-H, Dippold B, Dippold W (1994). Inhibition of anti-GD3-ganglioside antibody-induced proliferation of human CD8+ Tcells by CD16+ natural killer cells. Eur J Immunol.

[B44] Gosink MM, Petrie HT, Tsinoremas NF (2007). Electronically subtracting expression patterns from a mixed cell population. Bioinformatics.

